# Extracorporeal Cardiopulmonary Resuscitation (ECPR) for Out-of-Hospital Cardiac Arrest due to Pulseless Ventricular Tachycardia/Fibrillation

**DOI:** 10.1155/2020/6939315

**Published:** 2020-07-17

**Authors:** Konstantinos Dean Boudoulas, Bryan A. Whitson, David P. Keseg, Scott Lilly, Cindy Baker, Talal Attar, Quinn Capers, Richard J. Gumina, David W. Mast, Sree Veena Satyapriya, Dixie Davenport, Melinda Hazlett, Nahush Mokadam, Raymond Magorien, Ernest L. Mazzaferri

**Affiliations:** ^1^Division of Cardiovascular Medicine, The Ohio State University, Columbus, Ohio, USA; ^2^Division of Cardiothoracic Surgery, The Ohio State University, Columbus, Ohio, USA; ^3^Columbus Division of Fire, Columbus, Ohio, USA; ^4^Perfusion Services, The Ohio State University, Columbus, Ohio, USA; ^5^Department of Anesthesiology, The Ohio State University, Columbus, Ohio, USA; ^6^Ross Heart Hospital, The Ohio State University Wexner Medical Center, Columbus, Ohio, USA

## Abstract

**Background:**

Survival rates for out-of-hospital cardiac arrest are very low and neurologic recovery is poor. Innovative strategies have been developed to improve outcomes. A collaborative extracorporeal cardiopulmonary resuscitation (ECPR) program for out-of-hospital refractory pulseless ventricular tachycardia (VT) and/or ventricular fibrillation (VF) has been developed between The Ohio State University Wexner Medical Center and Columbus Division of Fire.

**Methods:**

From August 15, 2017, to June 1, 2019, there were 86 patients that were evaluated in the field for cardiac arrest in which 42 (49%) had refractory pulseless VT and/or VF resulting from different underlying pathologies and were placed on an automated cardiopulmonary resuscitation device; from these 42 patients, 16 (38%) met final inclusion criteria for ECPR and were placed on extracorporeal membrane oxygenation (ECMO) in the cardiac catheterization laboratory (CCL).

**Results:**

From the 16 patients who underwent ECPR, 4 (25%) survived to hospital discharge with cerebral perfusion category 1 or 2. Survivors tended to be younger (48.0 ± 16.7 vs. 59.3 ± 12.7 years); however, this difference was not statistically significant (*p*=0.28) likely due to a small number of patients. Overall, 38% of patients underwent percutaneous coronary intervention (PCI). No significant difference was found between survivors and nonsurvivors in emergency medical services dispatch to CCL arrival time, lactate in CCL, coronary artery disease severity, undergoing PCI, and pre-ECMO PaO_2_, pH, and hemoglobin. Recovery was seen in different underlying pathologies.

**Conclusion:**

ECPR for out-of-hospital refractory VT/VF cardiac arrest demonstrated encouraging outcomes. Younger patients may have a greater chance of survival, perhaps the need to be more aggressive in this subgroup of patients.

## 1. Introduction

There are at least 350,000 out-of-hospital cardiac arrests per year that occur in the United States of America. Pulseless ventricular tachycardia (VT) and/or ventricular fibrillation (VF) are the etiology in approximately one-fourth of cardiac arrests with a significant portion thought to be due to acute coronary syndromes; the overwhelming majority of these individuals will have refractory VT/VF or degeneration to nonperfusing rhythms, pulseless electrical activity (PEA), or asystole, resulting in mortality [1–4]. For these patients, innovative strategies have been developed from the field to the cardiac catheterization laboratory (CCL) for potential coronary artery revascularization in order to increase survival. Extracorporeal cardiopulmonary resuscitation (ECPR) programs using extracorporeal membrane oxygenation (ECMO) as an adjunct to cardiopulmonary resuscitation (CPR) have been developed and have shown encouraging outcomes in patients with out-of-hospital refractory VT and/or VF cardiac arrest, though data have been limited [4–15].

On August 15, 2017, The Ohio State University Wexner Medical Center and Columbus Division of Fire launched a collaborative ECPR program for out-of-hospital refractory VT and/or VF cardiac arrest. This article describes our experience and outcomes after the implementation of an ECPR program serving Columbus, Ohio. In addition, this article may potentially serve as a template for other institutions worldwide considering the initiation of such a program.

## 2. Methods

The Ohio State University Wexner Medical Center and Columbus Division of Fire collaboratively developed an ECPR program focusing solely on out-of-hospital refractory pulseless VT and/or VF cardiac arrest. The data presented are from August 15, 2017, to June 1, 2019. This retrospective study was approved by the Institutional Review Board of The Ohio State University.

### 2.1. Protocol Description: In the Field

**Figure 1:**
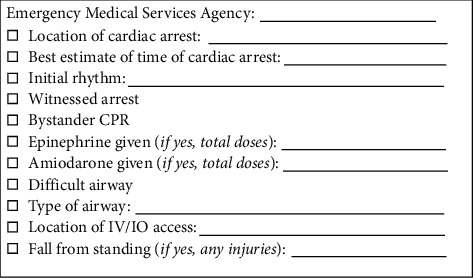
A handoff checklist used by the Columbus Division of Fire to assist in relaying pertinent information for patients being transferred from the field to our institution's cardiac catheterization laboratory as an extracorporeal cardiopulmonary resuscitation (ECPR) alert. CPR = cardiopulmonary resuscitation; IO = intraosseous; IV = intravenous.

Out-of-hospital cardiac arrest presenting rhythm had to be pulseless VT and/or VF upon arrival of *emergency medical services* (EMS) personnel. Moreover, VT and/or VF had to be refractory to three consecutive defibrillations (either 360 Joules monophasic or 200 Joules biphasic) in which the third defibrillation was a sequential defibrillation using 2 devices. Additionally, subjects had to be ≥18 to ≤65 years of age, cardiac arrest had to be witnessed, and bystander CPR had to be initiated to be considered for the ECPR protocol. Epinephrine and amiodarone were administered as per advanced cardiovascular life support (ACLS) protocol [16]. The patients were also intubated, and the automated CPR device (LUCAS chest compression system, Stryker Medical, Portage, Michigan) was placed and initiated at 100 to 120 beats per minute. Exclusion criteria in the field included *do not resuscitate* (DNR) prespecified wishes by the victim, asystole at any time, PEA upon arrival, and lack of automated CPR device in place for transportation. Subjects with cardiac arrest due to nonshockable rhythms were excluded from the ECPR protocol, as previously shown to have poor outcomes and/or survival (12). Once field criteria were met, an “*ECPR Alert*” was called to The Ohio State University Wexner Medical Center, which in turn activated a page to the ECPR team to assemble in the CCL in less than 30 minutes. The patient was emergently transferred directly to the CCL bypassing the emergency department. A handoff checklist (Figure 1) from the EMS personnel to the CCL team was developed to assist in relaying pertinent information.

### 2.2. Protocol Description: Cardiac Catheterization Laboratory

The ECPR team in the CCL included the following personnel: interventional cardiologist (with or without a fellow), cardiothoracic surgeon (with or without a fellow), anesthesiologist, cardiovascular perfusionist, CCL nurses, radiology technicians, pharmacist, and respiratory therapist. Personnel roles in the CCL were predefined in order to maximize efficiency and avoid confusion upon arrival of the patient. In addition, multiple mock patient drills were performed including with the Columbus Division of Fire prior to program initiation.

**Figure 2:**
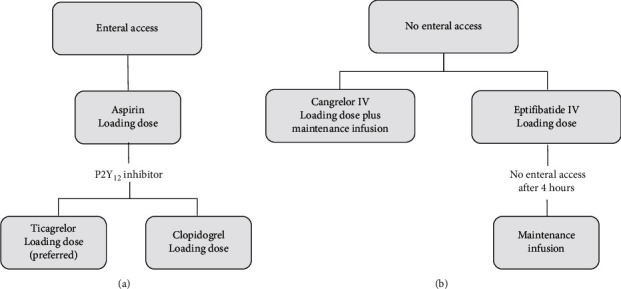
Algorithm for antiplatelet therapy for patients undergoing extracorporeal cardiopulmonary resuscitation (ECPR) and percutaneous coronary intervention based on the presence of (a) enteral access (e.g., nasogastric tube and oral gastric tube) or (b) no enteral access. IV = intravenous.

Upon arrival to the CCL, the LUCAS device was briefly paused to determine the patient's rhythm. If asystole was present, then the patient was pronounced deceased. If PEA was present, then a multidisciplinary discussion was held in the CCL to determine whether to proceed with the EPCR protocol; decision to proceed with ECPR was individualized based on available information including age, duration of cardiac arrest, and comorbidities. If pulseless VT and/or VF were present, defibrillation and ACLS were continued. An arterial blood gas was quickly obtained, which was also used to determine the inclusion criteria for placing the patient on ECMO. Based on the review of current literature of successful ECPR programs, inclusion criteria included lactate ≤12 mg/dL, end-tidal carbon dioxide (EtCO_2_) ≥10 mmHg, partial pressure of oxygen (PaO_2_) ≥50 mmHg, adequate peripheral access, and willingness of the patient to take blood products. Individuals that did not meet inclusion criteria in the CCL were pronounced deceased. Once inclusion criteria were met, femoral arterial and venous access were obtained followed by placement of ECMO cannulas (Medtronic, Minneapolis, Minnesota) under fluoroscopy (15 F or 17 F arterial cannula for a male; 15 F arterial cannula for a female; and 25 F multistage venous cannula for all patients). After ECMO (Getinge, Wayne, New Jersey) initiation, the LUCAS device was stopped. All subjects placed on ECMO underwent a diagnostic coronary arteriogram. Percutaneous coronary intervention (PCI) was performed when deemed clinically necessary using unfractionated heparin for anticoagulation, and specific antiplatelet therapy depended on the presence or not of enteral access, as described in Figure 2.

All attempts were made in the CCL to percutaneously place an antegrade 10 F pediatric femoral cannula, guided by fluoroscopy and ultrasound, in the superficial femoral artery on the side of the ECMO arterial cannula to perfuse the lower extremity. The antegrade catheter was configured as a “Y” circuit off the ECMO arterial tubing proximal to the femoral arterial cannula. In addition, targeted temperature (35-36°C) management for 24 hours was initiated via the ECMO circuit starting in the CCL.

**Figure 3:**
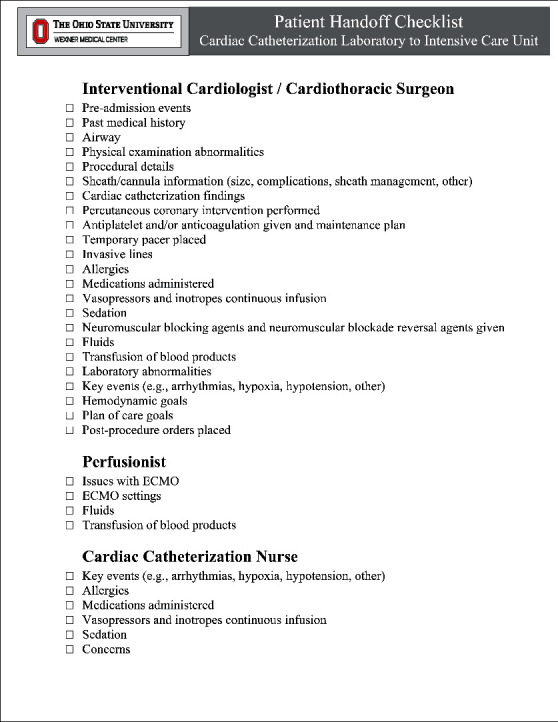
A handoff checklist used at our institution to assist in relaying pertinent information for extracorporeal cardiopulmonary resuscitation (ECPR) patients being transferred from the cardiac catheterization laboratory to the intensive care unit; ECMO = extracorporeal membrane oxygenation.

Once the patient was placed on ECMO and after cardiac catheterization, if the patient achieved return of spontaneous circulation (ROSC), they were transported to the intensive care unit (ICU) where a multidisciplinary team led by an intensivist cared for the patient. A handoff checklist (Figure 3) from the CCL team to the ICU team was developed to assist in relaying pertinent information. If the patient did not achieve a perfusing rhythm within 90 minutes of arrival to CCL despite ECMO, and PCI when indicated, then the patient was pronounced deceased.

### 2.3. Protocol Change

**Figure 4:**
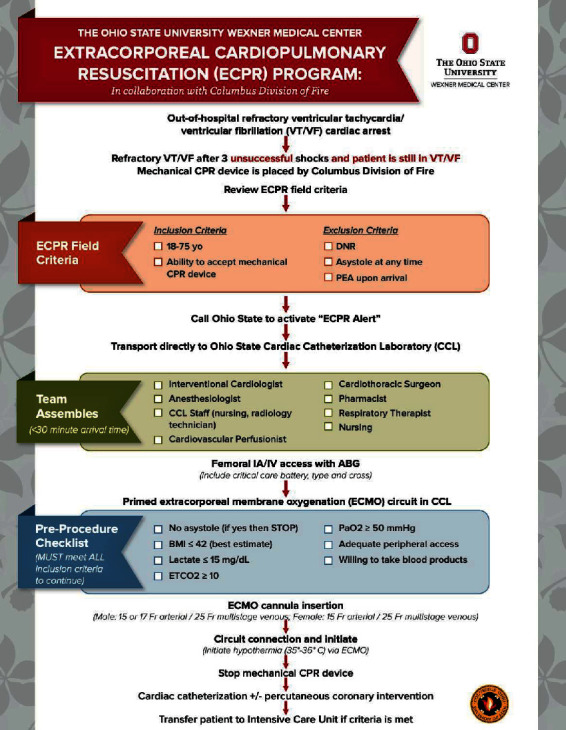
The Ohio State University Wexner Medical Center in collaboration with the Columbus Division of Fire extracorporeal cardiopulmonary resuscitation (ECPR) protocol for to out-of-hospital cardiac arrest due to refractory ventricular tachycardia and/or ventricular fibrillation. BMI = body mass index; CPR = cardiopulmonary resuscitation; DNR = do not resuscitate; ETCO_2_ = end-tidal carbon dioxide; IA = intra-arterial; IV = intravenous; PaO_2_ = partial pressure of oxygen; PEA = pulseless electrical activity.

In March 2018, after multidisciplinary review of our data, in-the-field inclusion criteria were changed to the following: age increased to 75 years, cardiac arrest did not have to be witnessed, and there was no longer a requirement for bystander CPR. In October 2018, CCL inclusion criteria were also changed to include presenting lactate of ≤15 mg/dL. These changes were implemented in order to provide this potentially lifesaving therapy to a wider population pool, as the available data at that time suggested the potential for favorable outcomes despite making these changes. The final version of the ECPR protocol is shown in Figure 4.

### 2.4. Study Population

From August 15, 2017, to June 1, 2019, there were 86 patients that were evaluated in the field for cardiac arrest by the Columbus Division of Fire EMS providers. Of these patients, 42 (49%) had refractory pulseless VT and/or VF after undergoing three defibrillation attempts as per the defined protocol and were emergently transported to the Ross Heart Hospital CCL as an “*ECPR Alert*”. From these 42 patients, 16 (38%) met the final inclusion criteria in the CCL (Figure 4) and were placed on ECMO; there was one patient that underwent ECPR on two separate time periods, October 2018 and April 2019, and these two ECPR events were analyzed separately.

### 2.5. Complications

Complications that occurred during hospitalization were determined. Complications included vascular (limb ischemia, bleeding, or dissection/rupture), new hemodialysis, infection (e.g., bacteremia, aspiration pneumonia, and urinary tract infection), stroke or seizure, severe anoxic brain injury, and bleeding from the gastrointestinal tract.

### 2.6. Statistical Analysis

Descriptive data are shown as mean ± 1 standard deviation. To determine statistical significance between the two groups Fisher's exact test, Student's *t*-test or Mann–Whitney test were used where appropriate. A *p* value <0.05 was considered as statistically significant.

## 3. Results

Demographics and other parameters for individual patients that met inclusion criteria for ECPR and placed on ECMO in the CCL are shown in Table 1. There were 4 of the 16 patients (25%) that survived to hospital discharge. In addition, all 4 of these patients survived to hospital discharge with cerebral perfusion category (CPC) 1 or 2 (Table 2). The remaining 12 patients died due to severe anoxic brain injury (*n* = 9), refractory cardiac arrest in the CCL (*n* = 1), aortic rupture in the CCL (*n* = 1), or predominantly multiorgan failure (*n* = 1).

Summary of the past medical history for the overall cohort and for the survivor and nonsurvivor subgroups that were placed on ECMO in the CCL are shown in Table 2. The past medical history was unable to be obtained in 4 of the 12 patients who did not survive. The mean age for the overall cohort was 56.4 ± 14.1 years. There was no statistically significant difference in the mean age between survivors and nonsurvivors (48.0 ± 16.7 years and 59.3 ± 12.7 years, respectively; *p*=0.28); however, there was a trend towards younger age in patients that survived, though this was not statistically significant most likely due to a small number of patients. EMS dispatch to on-scene arrival time for the overall cohort was 6.1 ± 2.2 minutes. There was no statistically significant difference in EMS dispatch to on-scene arrival time between survivors and nonsurvivors (4.5 ± 2.5 minutes and 6.7 ± 2.0 minutes, respectively; *p*=0.18). EMS dispatch to on-scene arrival times were relatively short due to having numerous fire stations and being strategically located throughout the city. EMS on-scene to CCL arrival time for the overall cohort was 38.1 ± 9.0 minutes. There was also no statistically significant difference in EMS on-scene to CCL arrival time between survivors and nonsurvivors (40.8 ± 6.7 minutes and 37.1 ± 9.7 minutes, respectively; *p*=0.43). EMS dispatch to CCL arrival time for the overall cohort was 44.2 ± 9.2 minutes. There was no statistically significant difference in EMS dispatch to CCL arrival time between survivors and nonsurvivors (45.3 ± 6.1 minutes and 43.8 ± 10.3 minutes, respectively; *p*=0.74) (Table 2). Laboratory values obtained in the CCL for the overall cohort and for the survivor and nonsurvivor subgroups are shown in Table 2.

Overall, 8 of 15 patients were found to have coronary artery disease (CAD) ≥50% stenosis on coronary arteriogram in a major coronary artery; there was no statistically significant difference in CAD ≥ 50% stenosis between survivors (*n* = 1) and nonsurvivors (*n* = 7) (*p*=0.28). Coronary arteriogram was not performed in 1 patient who did not survive due to aortic rupture. Overall, 6 of 15 patients who underwent a coronary arteriogram also underwent PCI with no statistically significant difference between survivors and nonsurvivors (*n* = 1 and *n* = 5, respectively; *p*=1.0) (Table 2).

Duration on ECMO and total hospital days for the overall cohort were 3.8 ± 2.2 and 8.1 ± 6.7 days, respectively. There was no statistically significant difference in duration of ECMO in survivors and nonsurvivors (4.8 ± 2.5 and 3.4 ± 2.2, respectively; *p*=0.38). Total hospital days were significantly greater in survivors as compared to nonsurvivors (15.5 ± 4.7 vs. 5.7 ± 5.4, respectively; *p* ≤ 0.05) (Table 3). Complications and number of patients with each complication are shown in Table 3.

## 4. Discussion

Survival rates for out-of-hospital cardiac arrest are very low, and neurologic recovery for those that survive is poor [1, 2]. It was suggested in 1966 that the heart-lung machine used for cardiac surgery could potentially be used as an extended form of cardiopulmonary resuscitation in select patients with cardiac arrest [17]. ECMO is an extracorporeal (i.e., outside of the body) technique that provides both cardiac and respiratory support (i.e., cardiopulmonary bypass) to sustain life [18]. By using ECMO as an adjunct to CPR, blood flow can be restored in patients with prolonged cardiac arrest in order to provide adequate perfusion, particularly cerebral perfusion which often is the cause of death in this patient population. The development of ECPR programs, incorporating ECMO as an adjunct to CPR, has shown encouraging results in patients with out-of-hospital cardiac arrest. Though limited studies, ECPR for out-of-hospital cardiac arrest has demonstrated survival to hospital discharge ranging from 4% to 45% with adequate neurologic recovery of CPC 1 or 2 [4–15]. On August 15, 2017, The Ohio State University Wexner Medical Center and Columbus Division of Fire launched a collaborative ECPR program for refractory pulseless VT and/or VF cardiac arrest to serve Columbus, Ohio, with promising outcomes. Our ECPR program resulted in survival to hospital discharge of 25% with all patients having a CPC 1 or 2 at the time of discharge.

Greater than 350,000 annual out-of-hospital cardiac arrests occur in the United States of America in which approximately one-fourth are due to VT and/or VF; a significant portion of these cases are believed to be due to underlying CAD and acute coronary syndromes. Thus, it has been postulated that prompt coronary artery revascularization in the CCL in these patients can reverse the underlying cause of cardiac arrest and possibly increase the chances of survival [1–4]. This has provided a potential ECPR target for patients with cardiac arrest due to VT and/or VF. Pozzi et al. [12] evaluated 68 patients (43 ± 11 years) that underwent ECPR for refractory out-of-hospital cardiac arrest; patients were divided into two groups: shockable and nonshockable rhythms. The shockable rhythm group had a survival to hospital discharge of 31%, while there were no patients that survived in the nonshockable rhythm group [12]. Although data suggest that ECPR can have favorable outcomes for in-hospital cardiac arrest due to PEA [19], since our protocol focused on out-of-hospital cardiac arrest, it was determined by our group to only include cardiac arrest due to VT and/or VF based on previous experiences [12, 15, 20]. In our study, 53% of patients were found to have significant CAD (≥50% stenosis in a major coronary artery) on coronary arteriogram with 40% of these patients requiring PCI, as also previously reported [14]. However, the etiology of cardiac arrest in addition to acute coronary syndromes was related to other underlying pathologies as well (Table 1). This study demonstrated that ECPR protocol may be applied to other underlying pathologies besides CAD. Whether ECPR would be more beneficial in certain underlying etiologies remains to be defined.

Presenting lactate levels have been shown to have a strong prediction of survival in patients undergoing ECPR. Dennis et al. [14] evaluated 37 patients (median age of 54 years with range 47–58) that underwent ECPR for refractory cardiac arrest; survival to hospital discharge was 35%. Survivors were found to have a significantly lower pre-ECMO lactate value (median 5.2 with range 4.1–10.1 mmol/L) as compared to nonsurvivors (mean 11.2 with range 8.6–15 mmol/L; *p*=0.01); logistic regression analysis demonstrated only pre-ECMO lactate to be predictive of mortality (OR: 1.35; CI: 1.06–1.73; *p*=0.01) [14]. In addition, Maekawa et al. [6] demonstrated in a multivariate analysis that pupil diameter on hospital arrival was the most powerful independent predictor of neurologic outcomes (adjusted HR: 1.39 per 1 mm increase; 95% CI: 1.09–1.78); a pupil diameter of <6 mm was shown to be the optimal cutoff point. In our study, survivors had a trend towards younger age; however, this did not reach statistical significance likely due to the small number of patients. In our experience, due to patient variability and due to variability of underlying pathology, not one value in one particular parameter can accurately predict survival. Inclusion criteria for ECPR are put in place as surrogate markers to assist in predicting prolonged hypoperfusion, hypoxia, and ultimately anoxic brain injury, as in the majority of cases it is the rate-limiting step in survival.

A key component to an ECPR program is providing effective and efficient chest compressions in order to adequately perfuse the brain and decrease the devastating risk of anoxic brain injury. Automated CPR devices have shown to improve cerebral blood flow compared to manual CPR; these devices are critical to have in the field, particularly when the ambulance is moving in order to perform adequate compressions and for the safety of EMS personnel [21–24]. Not having an automated CPR device in place by EMS prior to transporting the patient from the field to the CCL was exclusionary for participation in our ECPR program.

Complications related to ECMO are common. A few of the more common complications seen in patients on ECMO that have been reported include bleeding (5–79%), renal failure (30–58%), infection (17–49%), neurologic (10–33%), acute liver failure (27%), and/or limb ischemia (13–25%) [25]. Complications seen in our overall cohort undergoing ECPR are similar to previously reported and can be seen in Table 3. Having an awareness of potential complications and rapid management strategies when they do occur can improve outcomes. All patients in our cohort were managed in an ICU by a multidisciplinary team led by physicians formally trained in intensive care. Our ECMO program also supports a considerable number of patients yearly. In addition, we have a high procedural volume CCL that serves a large referral territory with experienced, high volume operators.

It is essential to have ongoing assessments of the ECPR program and to establish a quality assurance program. At our institution, routine multidisciplinary meetings were established, including with the Columbus Division of Fire, to discuss data, outcomes, quality, and operational issues allowing adjustments to the protocol as deemed appropriate. In addition, a review of cases was performed at routine conferences (e.g., morbidity and mortality, multidisciplinary meetings). As a result of these meetings, and review of our data and other-like programs, in March 2018 and October 2018, the ECPR inclusion criteria were carefully broadened in order to expand this potentially lifesaving therapy to more patients (Figure 4).

Due to the nature of the disease, limitations to this study exist. Due to the inability for randomization, all comers with cardiac arrest due to VT and/or VF were included regardless of underlying pathology, which may have impacted outcomes. In addition, though this protocol was designed to include both genders, females only represented approximately 6% of ECPR patients, possibly suggesting a lower incidence of sudden cardiac death in females, as previously reported [26, 27]. Further, there were several confounders that existed including variation in witnessed cardiac arrest status, bystander CPR, EMS transportation times, cardiac arrest time prior to initiation of ACLS, and CPR/ECPR duration, which may have impacted variation of results.

## 5. Conclusion

On August 15, 2017, The Ohio State University Wexner Medical Center and Columbus Division of Fire launched a collaborative ECPR program for out-of-hospital refractory VT/VF cardiac arrest demonstrating encouraging outcomes. Multidisciplinary collaboration is critical for a successful program. In addition to CAD, the underlying pathology of VT/VF cardiac arrest was found to be due to other pathologies, which can successfully recover. Younger patients may have a greater chance of survival, perhaps the need to be more aggressive in this subgroup of patients. The development of national and international registries will help to generate more data that may change how we approach this patient population.

## Figures and Tables

**Table 1:** Demographics and other parameters for patients placed on extracorporeal membrane oxygenation (ECMO) in the cardiac catheterization laboratory (CCL) as part of the extracorporeal cardiopulmonary resuscitation (ECPR) protocol.

Subject	ECPR date	Age (years)	Gender	EMS dispatch to CCL arrival (min)	EMS on-scene to CCL arrival (min)	PCI performed	Diagnosis	Discharged alive
1	9/15/17	68	Male	45	43	No	Acute myocarditis	Yes
2	12/11/17	52	Male	53	49	Yes	STEMI (LAD stent thrombosis)	Yes
3	4/15/18	54	Male	57	49	Yes	STEMI (LAD and LCx)	No
4	4/16/18	43	Male	54	45	No	Flail mitral valve leaflet	No
5^*∗*^	10/15/18	44	Male	38	34	No	WPW	Yes
6	11/3/18	68	Male	59	52	No	Aortic dissection/rupture	No
7	11/13/18	48	Male	49	44	No	Nonischemic CMP	No
8	11/16/18	28	Male	45	37	No	Cocaine overdose	Yes
9	11/30/18	74	Female	32	27	No	MVR 7 years prior with MVR 7 years prior	No
10	12/27/18	75	Male	46	40	Yes	STEMI (RCA)	No
11	1/3/19	61	Male	33	26	Yes	STEMI (LCx)	No
12	2/8/19	65	Male	39	28	Yes	STEMI (LAD)	No
13	2/19/19	66	Male	35	29	No	CAD with CABG 1 month prior	No
14	2/21/19	41	Male	36	32	Yes	STEMI RCA	No
15	3/4/19	72	Male	33	28	No	Cardiac amyloidosis	No
16^*∗*^	4/15/19	44	Male	53	46	No	Nonischemic CMP	No

CABG = coronary artery bypass grafting; CAD = coronary artery disease; CMP = cardiomyopathy; EMS = emergency medical services; LAD = left anterior descending artery; LCx = left circumflex artery; MR = mitral regurgitation; MVR = mitral valve replacement; RCA = right coronary artery; STEMI = ST elevation myocardial infarction; WPW = Wolff–Parkinson–White. ^*∗*^Same patient undergoing ECPR during two separate time periods, October 2018 and April 2019.

**Table 2:** Summary of clinical parameters for patients placed on extracorporeal membrane oxygenation (ECMO) in the cardiac catheterization laboratory (CCL) as part of the extracorporeal cardiopulmonary resuscitation (ECPR) protocol.

	Overall (*n* = 16)	Survivors (*n* = 4)	Nonsurvivors (*n* = 12)	*p* value
Age (years)	56.4 ± 14.1	48.0 ± 16.7	59.3 ± 12.7	0.28
Previously diagnosed hypertension (*n*)	6^*∗*^	2	4^*∗*^	1.00
Previously diagnosed hyperlipidemia (*n*)	4^*∗*^	1	3^*∗*^	1.00
Previously diagnosed diabetes mellitus (*n*)	1^*∗*^	0	1^*∗*^	1.00
Previously diagnosed CAD (*n*)	5^*∗*^	1	4^*∗*^	0.57
Previously diagnosed stroke (*n*)	0^*∗*^	0	0^*∗*^	1.00
Previously diagnosed heart failure (*n*)	3^*∗*^	1	2^*∗*^	1.00
History of smoking (*n*)	7^*∗*^	2	5^*∗*^	1.00
EMS dispatch to CCL arrival (min)	44.2 ± 9.2	45.3 ± 6.1	43.8 ± 10.3	0.74
EMS on-scene to CCL arrival (min)	38.1 ± 9.0	40.8 ± 6.7	37.1 ± 9.7	0.43
Lactate in CCL (mg/dL)	11.9 ± 2.6	11.2 ± 3.1	12.2 ± 2.5	0.61
Pre-ECMO PaO_2_ (mmHg)^*∗∗*^	80 (50–414)	75 (56–414)	84 (50–330)	0.71
Pre-ECMO EtCO_2_ (mmHg)	29.3 ± 13.1	26.5 ± 6.1	30.4 ± 15.2	0.50
Pre-ECMO pH (g/dL)	7.09 ± 0.13	7.05 ± 0.15	7.10 ± 0.13	0.53
Pre-ECMO hemoglobin (g/dL)	13.1 ± 2.7	13.6 ± 2.7	12.8 ± 2.8	0.63
Presence of CAD ≥ 50% seen during coronary arteriogram (*n*)	8^§^	1	7^§^	0.28
PCI performed (*n*)	6	1	5	1.0

CAD = coronary artery disease; EMS = emergency medical services; EtCO_2_ = end-tidal carbon dioxide; PaO_2_ = partial pressure of oxygen; PCI = percutaneous coronary intervention. ^*∗*^Past medical history unable to be obtained in 4 of the 12 patients who did not survive. ^*∗∗*^Median value used for PaO_2_ due to large standard deviation. ^§^Coronary arteriogram was not performed in 1 patient who did not survive due to aortic rupture.

**Table 3:** Survival, days on extracorporeal membrane oxygenation (ECMO), days in hospital, and complications for patients undergoing extracorporeal cardiopulmonary resuscitation (ECPR).

	Overall (*n* = 16)	Survivors (*n* = 4)	Nonsurvivors (*n* = 12)	*p* value
Survival to discharge (*n*)	4	4^*∗*^	0	—
Total days on ECMO	3.8 ± 2.2	4.8 ± 2.5	3.4 ± 2.2	0.38
Total days in hospital	8.1 ± 6.7	15.5 ± 4.7	5.7 ± 5.4	<0.05
Complications (*n*)				
Vascular (limb ischemia, bleeding, or dissection/rupture)	6	2	4	0.60
New hemodialysis	5	1	4	1.00
Infection	4	2	2	0.24
Stroke or seizure	4	2	2	0.24
Severe anoxic brain injury	9	0	9	<0.05
Bleeding from the gastrointestinal tract	1	1	0	0.25

^*∗*^All survivors were discharged with cerebral perfusion category 1 or 2.

## Data Availability

The retrospective data used to support the findings of this study are restricted by HIPPA and IRB in order to protect patient privacy. Data are available from the corresponding author for researchers who meet the criteria for access to confidential data.
